# Editorial

**Published:** 1886-02

**Authors:** 


### Editorial.

---

It is probable that no special branch of medical knowledge has increased more rapidly within the last quarter of a century than gynæcology. As is almost universally the result of any innovation in medicine or surgery, enthusiasts carry the new ideas to various excesses, till the medical mind, stimulated by the wail of the suffering patients, comes to a realization of the truth, and the innovation takes its proper place of importance among the tried and reliable facts of medical knowledge.

Calomel and venesection a few years ago suffered ostracism for the abuses they received at the hands of the profession, and only of late years are beginning to be looked upon as the best of their kind when indicated.

The number of text-books on gynæcology, and the quantity of current medical literature upon this subject, has been relatively much larger than that devoted to any other branch of medicine or surgery.

The improvements that have been made in uterine and abdominal surgery, due to this enthusiasm, are laudable and for the good of womankind. The abuses referred to consist in too prevalent over-treatment of the uterus for ailments ascribed to it, and with which it is in no way connected.

Ailments for which no readily assignable cause could be found were apt to be laid to the uterus. It is a disgrace to the profession that the uterus has been so indiscriminately cauterized, scraped, replaced and subjected to medication. The condition of the organ and the general health of the patients so treated now bear testimony to the evil that has been irreparably done, and the conscientious thoroughness with which the physician did his work. The methods of treatment are not to be wondered at when one considers the small amount of pathological knowledge of these affections which characterized the most prominent early text-books, and as exemplified in the practice of men eminent in this branch.

The general practitioner, from the beginner to the aged, in country and in city, has been led thereby to make diagnoses and treat women for uterine ailments without any adequate knowledge of the pathology or of the technique of gynæcological practice.

Cylindrical speculæ of several sizes, a uterine sound, dressing forceps, fuming nitric acid, a stick of lunar caustic, a bottle of Churchill's tincture of iodine, and cotton, constituted the armamentarium.

Ulceration of the os, leucorrhœa, metritis, etc., were all individual diseases, and were treated rather than the diseased action of which they were the result.

The largest of the speculæ the patient could bear was introduced, the sound passed carelessly into the uterus, and, as indicated, the stick of caustic, nitric acid, or other irritants, carried to the fundus.

One leader in gynæcology said sterility and dysmenorrhœa can be cured by dividing the cervix; forthwith, every woman thus suffering had her cervix slit open. Then, laceration of cervix became known as a factor in uterine pathology, and all torn cervixes were indiscriminately sewed up.

The time has now come when the extensive clinical experience of able observers has gradually worked a reform in their gynæcological practice, and the full statement of these rational deductions are being spread broadcast for the instruction of the general practitioner.

To be a good gynæcologist it is necessary that the physician be a good general practitioner. Otherwise he will probably be too narrow in his practice. He must comprehend the deep primary principles of uterine pathology, and instead of the harsh and painful modes of examination and treatment of the past, use the gentler, milder and less irritating methods of to-day. Appreciating the relations of local to constitutional conditions, often the uterus will escape treatment, when formerly it would have been vigorously handled.

On this subject, Dr. Emmet says: "We shall have made a great advance in solving the problem as to the true pathology of many supposed uterine diseases, when we seek for the cause outside of the uterine limits. For many years I have been convinced of the truth that we have been misled by confounding cause and effect."

As to the harm of introducing the sound into the uterine cavity, and of making intra-uterine applications, the same author says: "Unless a patient has suffered from bleeding directly due to the condition of the mucous membrane, and has needed some prompt application of an astringent, I have not made an application above the internal os in any private hospital since 1879, and had for some time before abandoned the method in my office practice, and my views have since been carried out by my assistants in the Women's Hospital. The result has been decidedly more satisfactory, with fewer relapses and a more rapid recovery in every case. As long as we continue to treat the recognized pathological changes in the uterus as the primary conditions, it will seem proper to apply agents directly, or as nearly as possible, to the parts involved. If, however, we appreciate the fact that some inflammatory change in the pelvic tissues, outside of the uterus, is existing, or has existed, in almost every instance of so-called uterine disease in the non-puerperal state, we shall not attempt to treat the effect by agents introduced into the cavity of the uterus, without being prepared for recurring attacks of cellulitis excited by our mode of practice, and not due to some accidental cause. If so-called ulceration of the cervix is accepted as a cause, and not as an effect, the use of caustic applications is a consistent practice, and should be persevered in till the surface is healed; but, if it is held that the increased secretion is simply an effort of nature to relieve an obstructed venous circulation, and that the erosion is a surface from which the epithelium has been washed away by the discharge flowing over it, then such a course of treatment is deemed to be not only irrational, but hurtful. It is believed that in time professional opinion can be influenced to abandon intra-uterine medication as one not founded upon sound views of pathology, and to recognize the different forms and shades of pelvic inflammation outside of the uterus, now usually overlooked, and constituting the chief factor in the diseases of women."

The practitioner who now practices gynæcology, in the light of the present knowledge of uterine pathology, can not fail to do infinitely less harm than his predecessors, and, at the same time, restore to health many heretofore incurable. By this practice, these cases, which have been an opprobrium to our art, in a great measure, can be made to disprove the ofttimes repeated saying: "Once begin uterine treatment, and you will be obliged to continue it."

